# Formation of medical student professional identity: categorizing lapses of professionalism, and the learning environment

**DOI:** 10.1186/1472-6920-14-139

**Published:** 2014-07-09

**Authors:** Walter Hendelman, Anna Byszewski

**Affiliations:** 1 Department of Cellular & Molecular Medicine, Faculty of Medicine, University of Ottawa, 451 Smyth Road, Ottawa, ON K1H 8M5, Canada; 2 Professor of Medicine, Director of Professionalism, undergraduate curriculum, Faculty of Medicine, University of Ottawa, Division of Geriatrics, The Ottawa Hospital, Affiliate Investigator, Ottawa Hospital Research Institute (OHRI), Ottawa, Canada

**Keywords:** Professional identity formation, Professionalism lapse, Learning environment, Student survey, Role modeling

## Abstract

**Background:**

Acquiring the values of medical professionalism has become a critical issue in medical education. The purpose of this study was to identify lapses in professionalism witnessed by medical students during their four year MD curriculum, and to categorize, from the students’ perspective, who was responsible and the settings in which these occurred.

**Methods:**

An electronic survey, developed by faculty and medical students, was sent to all students with two email reminders. It included quantitative responses and some open-ended opportunities for comments. All analyses were performed with SAS version 9.1.

**Results:**

The response rate was 45.6% (255 of 559 students) for all four years of the medical school curriculum. Thirty six percent of students had witnessed or been part of an exemplary demonstration of professionalism; 64% responded that they had witnessed a lapse of professionalism. At the pre-clerkship level, the most frequent lapses involved students: arrogance (42.2%), impairment (24.2%), followed by cultural or religious insensitivity (20.5%). At the clerkship level of training, where students are exposed to real clinical situations, the lapses involved primarily faculty (including preceptor and clinician) or other staff; these included arrogance (55.3%), breach of confidentiality (28.3%), and cultural or religious insensitivity (26.6%); impairment involved mostly students (25.5%). These findings are analyzed from the perspective of role modeling by faculty and in the context of the learning environment.

**Conclusions:**

Medical students witnessed a lapse of professionalism involving both fellow students as well as faculty and administrative staff, in several domains. Results from this study emphasize the importance of role modeling and the need for faculty development, to improve the learning environment. This study adds to the limited emerging literature on the forces that influence medical student professional identity formation.

## Background

In 1990 the American Board of Internal Medicine (ABIM) “established a project to enhance the evaluation of professionalism as a component of clinical competence and to promote the integrity of internal medicine.” Project Professionalism was launched in 1995 [1]. The report states: “Professionalism aspires to altruism, accountability, excellence, duty, service, honor, integrity and respect for others” and definitions are provided for each of these “elements of Professionalism.” The report also describes seven issues that “challenge or diminish the previously identified elements of professionalism”; these include: abuse of power, arrogance, greed, misrepresentation, impairment, lack of conscientiousness, and conflict of interest (acceptance of gifts, collaboration with industry). Robins et al [2] examined undergraduate (second year) medical students’ “perceptions of the ethical climate for learning” using open-ended questions. Their responses were analyzed using the ABIM taxonomy and the challenges to professionalism as defined, indicating the usefulness of this framework for studies of this nature.

Society expects that the medical profession will uphold the highest behavioral and ethical standards, which form the so-called ‘social contract’ [3]. Our task as medical educators should therefore be not only to provide the students with access to medical information, direction on how to use this medical knowledge, and the clinical skills necessary to become competent practitioners, but guidance on how to become medical professionals. It has been noted that “the values of the profession are becoming increasingly difficult for learners to discern” [4].

In retrospect, the cornerstone of Professionalism during the second part of the twentieth century is now recognized to have been role modeling as the major influence for the development of a professional identity. During the 1990’s, it became clear that due to the increasing diversity of our medical students, the changing paradigm of values held by society, along with the pressures of the marketplace, that the values and attributes of Professionalism do not necessarily accompany each and every student upon entry to medicine, and that these may not be nurtured optimally during the medical undergraduate years. Therefore it was proposed that the cognitive aspects of medical professionalism needed to be taught explicitly [5]. It is now recognized that part of the mission of each faculty of medicine must be the socialization to the core values of medical professionalism (see also [6]). Professionalism is now embraced by the LCME accreditation standard (MS-31-A) called ‘the learning environment’ [7], a standard which includes the values of Professionalism; this mandates an evaluation of the totality of influences within the whole medical curriculum and a comprehensive understanding of those factors influencing medical students.

The major recent initiatives to foster professionalism have focused on the development of these values in our medical students, particularly during the pre-clinical phase, in the formal curriculum, usually including a White Coat ceremony at the start of medical school. It is also understood that the explicit cognitive component needs to be reinforced by experiential learning [8]. In addition, considerable effort has gone into evaluating medical students in order to ensure that the objectives of this instruction have been achieved (e.g., [9]).

In our Faculty of Medicine, the students are first exposed to our Declaration of Professionalism [10] and the values (also called attributes) of Professionalism incorporated in this document during the orientation period. This is followed by a White Coat ceremony during which the students collectively ‘profess’ this Declaration in front of faculty, family and friends, and receive their own official copy of the document. During the pre-clerkship phase, four small group professionalism case-based sessions are held (two in each year) moderated by a faculty member. The introductory module to the clerkship, at the beginning of third year, includes a ‘re-affirmation ceremony’ for the class, with the emphasis on professionalism behavior. The assessment of our students throughout the four years includes professionalism behavior, and the regulations provide for remediation or other consequences for lapses.

Medical students are ‘taught’ by a village of doctors, residents and allied health personnel. Most medical educators are of the opinion that it is the hidden curriculum, as defined by Hafferty [11,12], particularly as students engage in their clerkship in the clinical setting, which undermines the efforts to instill the values of the medical profession (e.g., [13]).

One way of answering the question regarding the efforts and effectiveness of the professionalism curriculum is to collect the students’ observations and experiences. We report on a survey of medical students which was undertaken at the Faculty of Medicine of the University of Ottawa as part of the review of the professionalism program which had been in place for five years. The results of the survey regarding the program itself were reported previously [14]. A separate part of the survey included a question regarding witnessed exemplary professionalism behavior, and questions about the specifics of the unprofessional behavior, called lapses, across eleven domains. The electronic survey was designed to identify by category who was involved (student, tutor, clinician, preceptor, nurse, administrative staff) as well as where this event occurred (e.g., classroom versus clinic). This study offers a unique opportunity to gain insight into the real world of professionalism throughout the medical curriculum, as seen through the lens of the students themselves.

### Objective of study

To identify the nature of lapses in professionalism witnessed by students, to categorize who was involved and to identify the setting in which this occurred. This information can guide measures to improve the learning environment.

## Methods

**Figure 1:**
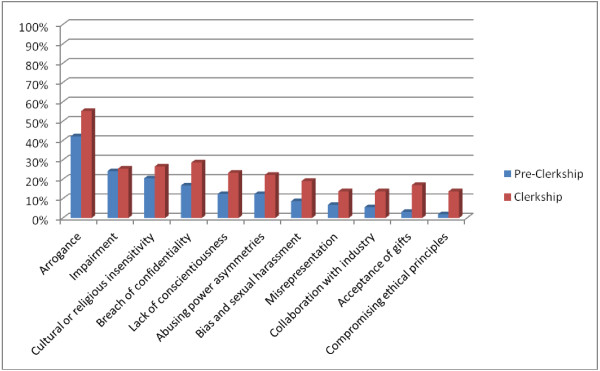
Percentage of students observing lapses (pre-clerkship vs clerkship).

The survey was developed by a small task group consisting of two medical students, with a faculty member (WH), and was subsequently approved by the committee responsible for review of the professionalism curriculum. The survey question regarding lapses of professionalism observed by the students were drawn from the “challenges” – called domains in this paper – outlined in the Project Professionalism report ([1], see also [2]). These include (Figure 1): arrogance, impairment, breach of confidentiality, lack of conscientiousness, abusing power asymmetries, misrepresentation, bias and sexual harassment, collaboration with industry, acceptance of gifts, and compromising ethical principles, to which was added cultural or religious insensitivity.

The survey questions were electronically set so that once the respondent indicated that a lapse had occurred in one of these domains, she/he could not proceed further until the information was provided as to where this incident had occurred (academic or clinical setting), and the identity of the source of the lapse in professionalism, the person involved in the incident: tutor (involved in small group teaching sessions), faculty member (could be any physician encountered on the ward), preceptor (the assigned supervisor of a small group of students on the clinical rotation), clinician, nurse, student, administrative staff, other hospital staff. The student respondent could indicate one or several lapses in each domain before proceeding to the next item.

The electronic survey, including quantitative responses and some open-ended opportunities for supplemental or additional comments (in both English and French), was sent out in December 2006 with two email reminders. The Ottawa Research Ethics Board had no objections to the content of the survey. The survey was not piloted, given the time pressures of the curriculum review being undertaken at the time at the Faculty of Medicine.

### Data analysis

Most of the questions were phased into 4 response levels which were collapsed into two overall categories: agree and disagree. For the analysis, the classification of clinician, tutor, faculty member and preceptor was renamed physician/teaching faculty. Administrative staff/other also included hospital (non-clinical) staff.

All analyses were performed with SAS version 9.1 (SAS Institute Inc., Cary, NC, USA.). Chi-square test and Fisher’s exact test (for cell count less than 5) were used to get p-values for categorical variables when comparing between pre-clerkship (years 1 and 2) and clerkship (years 3 and 4), when the curriculum changes from the classroom driven setting to a clinical milieu.

We defined the significant level as p < 0.05.

## Results

The response rate was 45.6%, with 255 responses from a possible 559 student population. The breakdown was: in year 1, 29% (74/152), in year 2, 34% (87/138), in year 3, 14% (36/135) and in year 4, 23% (58/134).

### Witnessed professionalism behavior

In total, 64.3% of the students (164 of 255) had witnessed a lapse of professionalism at some point of their training (Table 1), and this was more evident in the clerkship (72.3%, 68 of 94), compared to the pre-clerkship (59.6%, 96 of 161).

**Table 1:** Witnessed a lapse of professionalism as an MD student

	**Pre-Clerkship (n = 161)**	**Clerkship (n = 94)**	**Total (n = 255)**	**P-value 0.043**
Yes	96 (59.6%)	68 (72.3%)	164 (64.3%)	
No	65 (40.4%)	26 (27.7%)	91 (35.7%)	

When asked about their experience with professionalism, 38% of students had witnessed or been part of an exemplary demonstration of professionalism during their training. Some examples of these are included in Table 2.

**Table 2:** Exemplary professionalism – student commentaries

1.	School administrator who treats students with respect and offers sound advice and guidance…(*year 1 student*)
2.	Those who introduce students to patients and make sure the patient is aware of the opportunity they are giving to the student… (*year 2 student*)
3.	Some classmates by working hard, being honest, punctual, good listener…(*year 3 student*)
4.	A preceptor who willingly fills out evaluation forms…(*year 3 student*)
5.	A plastic surgeon…cares about his patients, …is giving back by being involved with various committees and teaching…(*year 3 student*)

### Types of lapses

The incidence of lapses across the 11 domains are shown for both the pre-clerkship and the clerkship together (Figure 1). Of those students who witnessed a lapse of professionalism, the six most frequent categories reported at the pre-clerkship level were: arrogance (42.2%), impairment (24.2%), cultural or religious insensitivity (20.5%), breach of confidentiality (16.8%); and both lack of conscientiousness and abusing power asymmetries ranked fifth (12.4%). At the clerkship level, the six most frequent categories of lapses of professionalism were the same but the rank order of the type of incidences changed: arrogance (55.3%), breach of confidentiality (28.7%), cultural or religious insensitivity (26.6%), impairment (25.5%), and lack of conscientiousness (23.4%); abusing power asymmetries was slightly less (22.3%). It should be noted that bias and sexual harassment was not far behind (19.1%) at the clerkship level, whereas it was infrequently reported (8.7%) at the pre-clerkship level. In both groups, the least frequent lapses included misrepresentation, collaboration with industry, acceptance of gifts and compromising ethical principles.

### Who was involved

**Figure 2:**
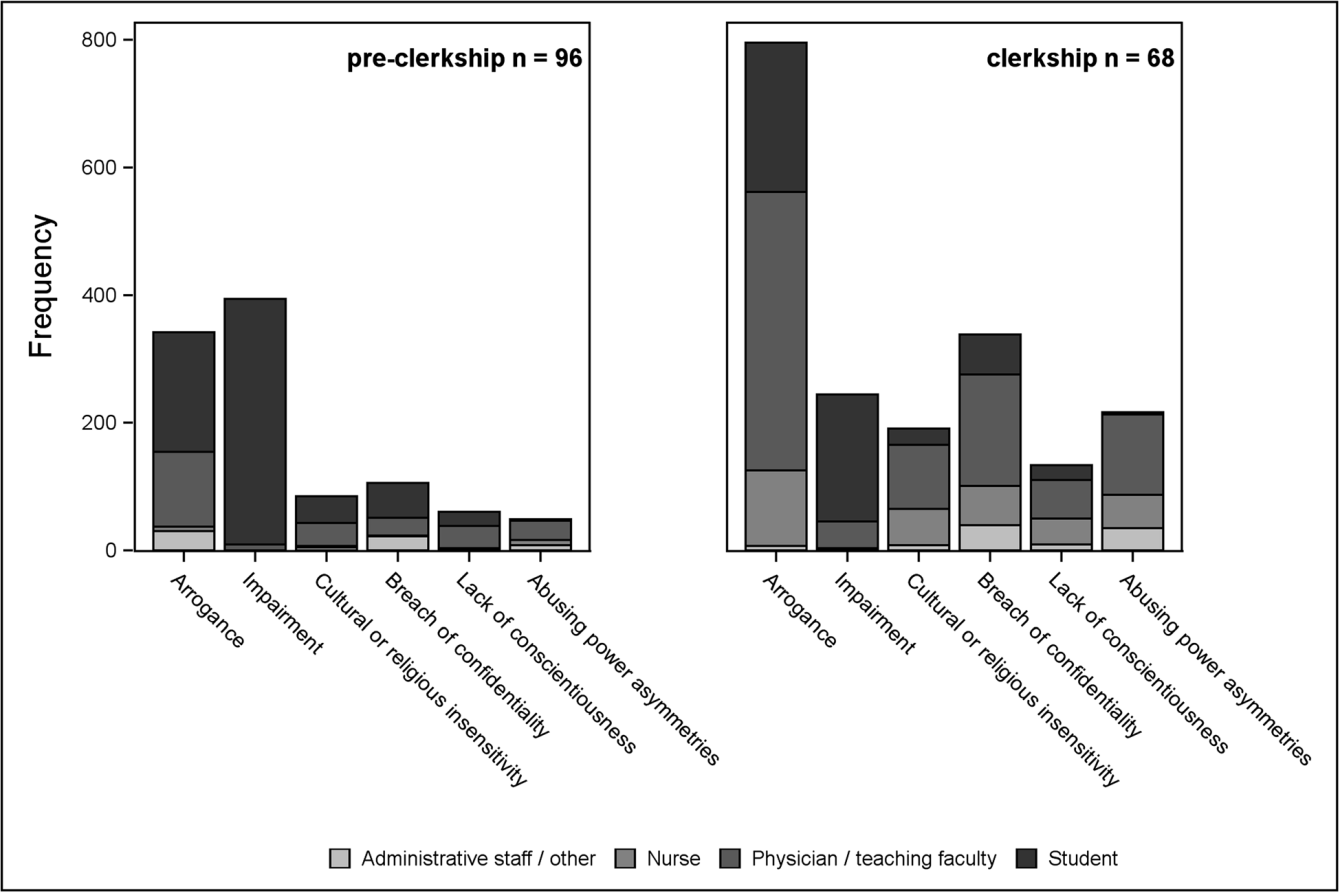
Who was involved – Lapses of Professionalism: pre-clerkship and clerkship.

Once the student respondent clicked to report a lapse in a domain, the set-up of the electronic survey allowed any number of incidences to be reported within that domain. The total number of reported incidences by the 164 students who witnessed a lapse of professionalism was 3,275 (approximately 20 per student overall). Arrogance was the most frequently reported lapse (1138 incidences), impairment the next most frequent (638), breach of confidentiality was next (445 incidences), followed by cultural or religious insensitivity (276) and abusing power asymmetries (266).The data for who was involved for pre-clerkship and clerkship is shown in Figure 2 for the six domains with the highest number of responses.

#### Pre-clerkship (Figure 2)

For the 96 students who reported lapses at the pre-clerkship level, there were 1,096 incidences, averaging approximately 10 per student.

Impairment was the most frequently reported lapse (394) and almost all of the reported incidences in this domain (>97%) involved students. The next most frequent lapse was arrogance, (342), with 187 reported incidences involving students (57% of this domain) and 117 (34%) involving physician/teaching faculty. Breach of confidentiality was also an issue (106 incidences) with 54 (51%) involving students and 28 (26%) involving physician/teaching faculty. Cultural or religious insensitivity (85 incidences) involved both students (49%) and physician/teaching faculty (42%), while lack of conscientiousness (61 incidences) involved largely faculty (57%) and students (36%). Abusing power asymmetries (49 incidences) involved mostly physician/teaching faculty (61%), as well as nurse (16%) and administrative staff/other (18%).

In summary, for the most frequent professionalism lapses at the pre-clerkship stage, students recognized that by far their student peers were the most common person implicated in lapses involving impairment, arrogance, and breach of confidentiality. Both students and faculty were implicated in lapses relating to cultural or religious insensitivity, while physician/clinical teacher and others were implicated in abusing power asymmetries.

#### Clerkship (Figure 2)

For the 68 students reporting lapses at the clerkship level, there were 2,179 incidences, averaging approximately 32 per student.

Arrogance was the most frequently reported lapse (796 incidences) with the majority attributed to physician/teaching faculty (55%), but with both students (29%) and nurses (15%) also being involved. Breach of confidentiality was a significant issue, with 339 reported incidences involving all: physician/teaching faculty (51%), students (19%), nurses (18%) and administrative staff/other (12%). Impairment was reported less often (244 incidences), but again students were the most frequently involved (81%). Abusing power asymmetries was also a significant issue (217 incidences) with physician/teaching faculty most often involved (57%), while nurses (24%) and administrative staff/other (16%) also contributing to this issue. Cultural or religious insensitivity was also present in the clinical environment (191 incidences), with all categories involved to some degree: physician/teaching faculty (52%), nurses (30%), students (13%), with some administrative staff/other (5%). Lack of conscientiousness (134 incidences) was seen by the students to involve all to some degree: physician/teaching faculty (45%), nurses (30%), students (17%) and administrative staff/other (7%).

In summary, in clerkship, where students are exposed to real clinical situations on the wards, the reported incidence of lapses increased in number and changed as to the most significant issues, as well as who was seen as the person responsible. The physician/clinical teacher in the form of preceptor, tutor or clinician were the most likely to have been involved in lapses involving arrogance and abusing power asymmetries. Breach of confidentiality and cultural or religious insensitivity involved all to some degree. Impairment for the most part was still likely to involve mostly students, similar to the trend seen in pre-clerkship, while arrogance remained an issue involving mainly students.

Comparing pre-clerkship to clerkship, the reported issues shifted to some degree because of the clinical environment, but nevertheless the top six issues remain the same at both stages.In the domains with low reported number of incidences (not shown in Figure 2) the reported incidence of bias and sexual harassment was higher at the clerkship level, with 36 incidences (for the 68 students); the majority (23, 64%) involved physician/teaching faculty and others involving both students (17%) and administrative staff/other (14%). At the pre-clerkship level, most reported incidences in this domain (20 for the 96 students) involved students (20, 60%) and the remainder (8) physician/teaching faculty. Acceptance of gifts, compromising ethical principles, collaboration with Industry, and misrepresentation were reported very infrequently.

Table 3 includes some sample comments from students in answer to the question as to how professionalism can be improved in our faculty. These suggestions include insights as to how to strive for improving the behavior of both students and clinical staff alike.

**Table 3:** How can we improve? – student commentaries

1.	I find it unprofessional of other students who play games or are on MSN the whole time during class or small groups…(*year 1 student*)
2.	…discuss issues where unprofessional conduct is noted and in such a way that the person is not necessarily pointed out, but that all learn from the issue…(*year 2 student*)
3.	More information on current rules dictating behaviour and the structures to enforce them, if I witness a breach of professionalism who do I contact? …(*year 2 student*)
4.	Need to expect and promote professional behavior in other areas of medical education, not just the professionalism seminars we receive twice a year…(*year 2 student*)
5.	Teach medical students that they are not the centre of the universe, some begin clerkship thinking nurses are there to serve them and that they will be giving orders. Teach them to value the opinions of physiotherapists, occupational therapists, nurses, nutritionists, social workers. (*year 3 student*)
6.	I feel there are certain times when preceptors may lack some respect with certain patient populations…( *year 3 student*)

### The setting

As expected, the site of occurrence of incidences at the clinical stage was generally in the hospital setting, on the wards. It is not completely clear where the incidences involving fellow students occurred, whether within the educational environment or during non-scheduled events outside the medical school.

## Discussion

This study adds to the emerging literature on medical students’ experience with observed lapses of professionalism, including not only on the type of lapses but also on the frequency of incidences and a comparison of pre-clerkship to clerkship. We also examined the specific situations where these lapses occurred, namely the nature of the incident, the ‘category’ of the person who was perceived to be the involved, and where the incident occurred. To our knowledge this type of information has not been reported.

We found there were definite differences of the learning environment in transition from pre-clerkship to the clerkship phase. In our study, almost 60% of students in the pre-clerkship had witnessed a lapse of professionalism and that number rose to 72% in the clerkship, whereas only 38% of students had witnessed or been part of an exemplary demonstration of professionalism during their training.

Our results indicate that a significant degree of student impairment and arrogance are prevalent in the pre-clerkship and that these behaviors continue into the clerkship. Thus, notwithstanding the emphasis on professionalism in the curriculum, it appears that there is a failure to alter some facet of the behavior of many of the students. In the clerkship phase of the curriculum, our study results showed a difference in the frequency of occurrence of the various types of lapses, as well as the person involved; a large proportion of the lapses were seen to involve physician/teaching faculty, nurses, as well as administrative staff/other.

Our results are similar to reports of the challenges students encounter in developing their professional identity, starting with one of the watershed articles “Ethics in a Short White Coat” [15]. Some of the nature of these student dilemmas was also elaborated by Hicks et al [16]. Studies conducted at the University of Chicago have shown that within months students in both their first year and in their third year tolerate situations which were initially thought to be unprofessional [17,18].

Previous reports by student themselves [19-23] further substantiate the negative perception of professionalism by the student body. According to these articles, medical students, residents, and junior doctors look up to their supervisors for role modeling and exemplary behavior as they embark in developing their own professional identity. The students have clearly indicated that the efforts to ‘teach’ Professionalism have in fact failed because of their interaction with their teaching faculty. In a study of how medical education had fostered or hindered the conceptions of compassion, altruism, and respect for patients [24], one student commented as follows: “This class is a great example of how this topic has been shoved down our throats too many times”.

Similar to other programs our students enter their clerkship in their third year after an introductory module to clinical work in the hospital. As is well recognized, it is during this clinical phase that the hidden curriculum exerts its greatest impact; we are using the term as defined by Hafferty [12] as “a set of influences that function at the level of organizational structure and culture”. The educational milieu changes to one of ward efficiency and ward hierarchy [22,25]. We need to prepare students for this shift of culture, where lapses of professionalism can occur as health care professionals try to work collaboratively in a medical environment challenged by complex health care system issues such as limited resources. The idealism and reason why students have entered medicine can be undermined, especially in the more senior years, by the actual behavior of those around the students, including a lack of sensitivity of their teachers [26]. “The devil is in the third year” has become a descriptive phrase, first used in a lead article by Hojat et al [13]; they demonstrated that a significant decline of empathy occurs in the third year, sadly at a time when there is a shift to patient care activities in most medical schools.

Much has also been written about the effect of the clinical environment on the development of student cynicism [27-29], which likely contributes to the erosion of professional identity formation. A study of third year clerks found that the most common transgressions of professionalism included “the use of derogatory language towards other services or patients and the disrespectful treatment of others” [30]. It is possible that this behavior could be responsible for the reports of the use of derogatory and cynical humor towards patients by students [31], residents and attending doctors [32,33]. The fact that there is often an absence or a major decline of any formal ‘teaching’ on Professionalism in the clerkship phase [34] sends a message to the students that this is no longer an important issue for them; some have termed this the null curriculum [35]. This is exactly the time when clinical challenges form the foundation for developing a professional identity. This view is supported by a recent systematic review which emphasizes that it is the clinician encounters that exert the greatest influence on students’ learning of professionalism [36].

Stern has compared the values taught in the formal curriculum to the values and behaviors that occur on the wards or clinics and has found that the latter are often contrary to the values we try to instill in the pre-clerkship phase [37]. It seems that the values of professionalism are also regularly conveyed in the so-called informal curriculum by residents while the students are on call, commonly at night, often when the staff person is not present [38]. It is noteworthy that there has not been a recognition of the role of residents in the discussions of the climate of professionalism during clerkship.

Our Faculty has introduced some initiatives with the potential to enhance professionalism, such as the creation of an Office of Professionalism and the coordination of the approach to professionalism issues in the pre-clerkship and clerkship with the hospital and other clinical milieus (see Table 4). The Faculty is working with affiliated institutions to align policies and procedures to deal with lapses of professionalism by students, teaching faculty, residents, nurses and allied health professionals, as well as non-clinical staff, and to address egregious or repeat offenders. Measures are also being taken to teach, evaluate and remediate the professionalism of residents (e.g., [39]). At the present time much of the emphasis of the professionalism movement is on student behavior [19,40,41]. New policies and procedures are being developed to address what should ensue when students (and residents) witness unprofessional incidents involving their preceptors and other health professionals while caring for patients. This has led to the introduction of a ‘concern/incident’ form. This form allows anyone in the faculty, including students, to report to the Vice-Dean of undergraduate medical education any incident of unprofessional behavior involving anyone – with complete confidentiality; the Vice-Dean then must investigate and deal with this report Our e-portfolio program [42] has evolved as a venue to develop professional identity. Other measures to enhance professionalism include faculty development workshops and awards celebrating the professionalism of students and faculty. To address some of the contributory elements to the hidden curriculum, we have adopted a “Complementary Approach” to Professionalism encouraging positive role models and establishing a staged approach to increase awareness and “guided interventions” [43].

**Table 4:** Steps to address professionalism and enhancement of the learning environment

●	Office of Professionalism (led by a clinician)
	○ Coordination of efforts of the Faculty of Medicine and the teaching hospitals
●	A Faculty of Medicine Policy on Medical Professionalism:
	○ Core Professionalism values
	○ Principles and Procedures
●	Professionalism sessions for students during pre-clerkship and clerkship:
	○ Small group case based discussions
	○ Experiential activities (reflective exercises, e-Portfolio)
●	Student feedback on the professionalism of the teaching faculty, residents and others (nurses, allied health) – mandatory after each clinical rotation:
	○ Concern/Incident form
	○ Formal evaluation of the learning environment
●	Consequences and staged interventions
	○ Process for remediation for students, faculty, residents and staff.
●	Faculty development workshops with an emphasis on the importance of role modeling
●	Awards celebrating Professionalism demonstrated/kudos letters

We seem to have come full circle to role modeling as the key to the development of professional identity formation; Kenny et al [44] have called this “an essential but untapped educational strategy”. Using similar phrasing, Cruess et al [45] have termed role modeling “the most powerful teaching strategy”. The qualities of role models and their part in the development of good doctors has been reviewed by Paice et al [46]. If role modeling is as important as indicated, a vigorous program of faculty development is necessary for the transformation of the environment where future physicians train, according to Steinert et al [47]. Glicken and Merenstein [48] have called this “educator professionalism”, the professionalization of medical educators as teachers. Birden et al [36] conclude their systematic review regarding the teaching of medical professionalism with the view that role modeling and personal reflections ideally guided by faculty are the most important elements in the teaching program.

Beyond addressing student and faculty (and residents) professionalism, some institutions have set out to transform the culture of professionalism throughout the whole faculty, namely the University of Texas Medical Branch in Galveston [49], the Indiana University School of Medicine [50,51], and the University of Chicago Pritzker School of Medicine [19], amongst others (e.g., The Mayo Clinic, [52]). Without this institutional support, it may not be possible to change the climate of professionalism. Bryden et al [53], using focus groups of faculty members at one institution, explored the teaching and evaluation of professionalism. The inquiry indicated that these faculty members felt powerless to address both mild and moderate lapses in professionalism, including their own and amongst their peers, “in the face of professional, departmental and institutional apathy.” In a study of residents and faculty at our institution, using concept mapping methods, barriers to reporting unprofessional behavior were identified; these include fear of repercussions, lack of incentive to report, and the perception that the ‘red tape’ and complex documentation are “not worth the effort when there are no consequences to the unprofessional physician” [54].

There are some limitations to this study, including that it was conducted at one institution, and the rather low response rate. In fact, analysis indicated that 326 students of the total student population of all four years (559) attempted the survey whereas only 255 students actually completed it; this is because the students were able to exit the survey prior to its completion. The fact that the response rate was less in the senior years was probably partly due to the fact that many of our clerkship (third-year) students were too involved in their clinical duties, and that the fourth-year students were engrossed with seeking their residency placements. More responses from this group might have given us valuable insights. In addition, we did not define some of the key behavioral terms, such as arrogance and impairment. For example, at the pre-clerkship phase, impairment might have meant the behavior that occurred at ‘celebrations’ following the examinations; during clerkship, impairment can be caused by the lack of sleep and self care due to stress that students endure in the work-intensive clerkships (such as internal medicine and surgery).

### Summary

In summary, our results suggest that we need to ensure the physicians of tomorrow have an opportunity to develop a solid professional identity, with exemplary role models. Some measures to enhance student and faculty professionalism have been taken since the survey and others are underway or proposed; these together with an effective assessment of professionalism of students, faculty and residents (and nurses and staff) emphasizing remediation, can assist in the transformation of the environment where future physicians train. As noted, very strong institutional support from the Dean, the Faculty of Medicine, the clinical (hospital) departments, as well as all the clinical venues where medical students train is required. In the future, this or a similar survey may be repeated when additional measures have been put in place, to assess the impact on the learning environment.

At a recent gathering (called a think tank), experts in the field of medical professionalism called for the development of “best practices informed by evidence-based research” including the prevalence, frequency and situations where unprofessional behavior occurs, as well as a question concerning the formation of professional identity [55]. The present study is a contribution to that literature.

## Conclusions

1. Lapses of professionalism involving students and clinicians, as well as nurses and staff, occur regularly, particularly in the clinical setting.

2. Role modeling of professionalism in all settings (including hospitals, clinics and other milieus) is the key component to address the challenge of the development of a positive medical student professional identity.

3. Additional measures, including a formal Office of Professionalism, feedback mechanisms, faculty development workshops, and remediation efforts are needed to address the improvement of the learning environment.

## Competing interests

The authors declare that they have no competing interests.

## Authors’ contributions

Both authors have made significant contributions to the article. WH and AB led the conception and design of the study and acquisition of the data, guided the data analysis, and drafted and revised the article. Both authors read and approved the final manuscript.

## Pre-publication history

The pre-publication history for this paper can be accessed here:

http://www.biomedcentral.com/1472-6920/14/139/prepub
